# Erratum to “Inhibition of Endoplasmic Reticulum Stress is Involved in the Neuroprotective Effect of bFGF in the 6-OHDA-Induced Parkinson’s Disease Model”

**DOI:** 10.14336/AD.2022.0727

**Published:** 2023-04-01

**Authors:** Pingtao Cai, Jingjing Ye, Jingjing Zhu, Dan Liu, Daqing Chen, Xiaojie Wei, Noah R Johnson, Zhouguang Wang, Hongyu Zhang, Guodong Cao, Jian Xiao, Junming Ye, Li Lin

We have noticed inadvertent errors in our article published in the August 2016 issue of Aging Dis (2016, 7(4): 336-449). The images of Figure 3C have been presented incorrectly. We have attached corrected Figure 3. The errors do not change the scientific conclusions of the article. The authors would like to apologize for the errors and any inconvenience caused.

**Figure 3.**
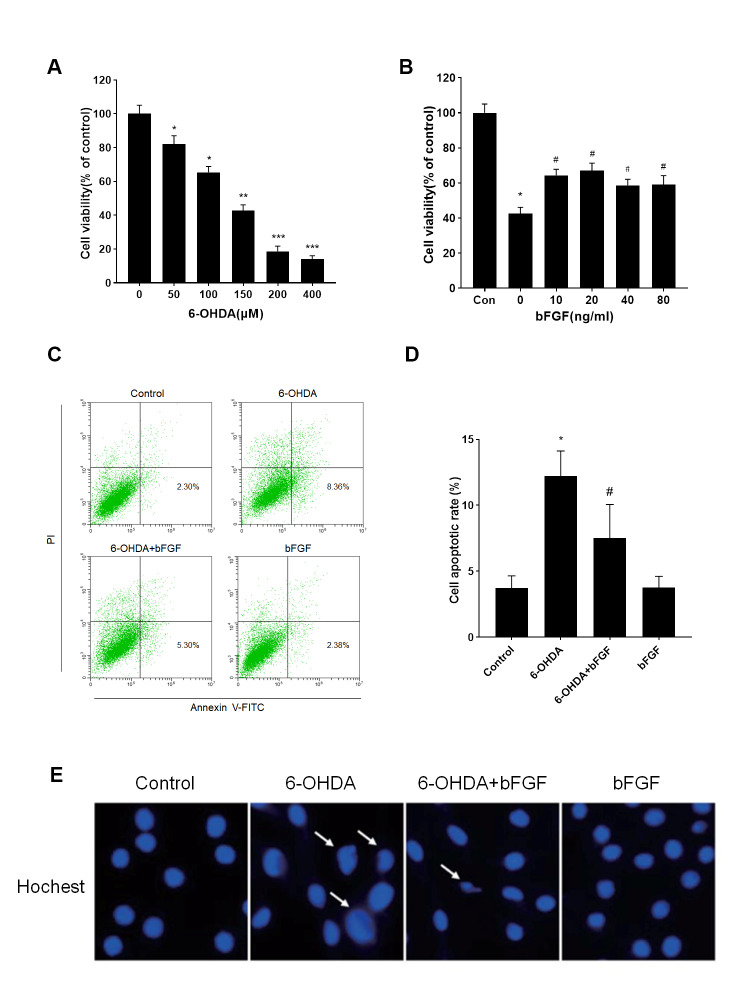
Effects of bFGF on 6-OHDA-induced apoptosis in primary hippocampal neurons. (A) Primary hippocampal neurons were treated with different concentrations of 6-OHDA for 24 h, and then cell viability was assessed by MTT assay. (B) Primary hippocampal neurons were treated with 6-OHDA (150 µM) and different concentrations of bFGF for 24 h, and then cell viability was assessed by MTT assay. (C) Primary hippocampal neurons were treated with 6-OHDA (150 µM) and bFGF (20 ng/ml) for 24 h, and then cells were stained with annexin V-FITC/propidium iodide and detected by flow cytometry; the lower right panel indicates the apoptotic cells. (D) Levels of cell apoptosis. (E) Hoechst staining of primary hippocampal neurons. ^*^P < 0.05 versus control group, ^**^P < 0.01, ^***^P< 0.001, ^#^*P* < 0.05 versus 6-OHDA group (n = 3).

